# Using a multimethod approach to develop implementation strategies for a cervical self-sampling program in Kenya

**DOI:** 10.1186/s12913-017-2160-0

**Published:** 2017-03-21

**Authors:** Irene Podolak, Caroline Kisia, Gloria Omosa-Manyonyi, Jarold Cosby

**Affiliations:** 1 Brock University, 213-2300 Upper Middle Road W., Oakville, ON L6M 0T4 Canada; 2 Action Africa Help International, Fawe House, Ground floor, Chania Avenue, P.O. Box 76598-00508, Nairobi, Kenya; 3 University of Nairobi, Uhuru Highway, Nairobi, 00100 Kenya; 4 Brock University, 500 Glenridge Ave, St. Catharines, ON L2S 3A1 Canada

**Keywords:** Participatory action research, Scenario based planning, Multimethod research, Informed decision-making, Self-sampling, Cervical cancer screening

## Abstract

**Background:**

Numerous health policy makers/researchers are concerned about the limitations of research being applied to support informed decision/policy making and the implementation of practical solutions. The aim of the Chaguo Letu project (which means our choice in Swahili) was to determine how local decision makers could apply a multimethod approach to make strategic decisions to effectively implement a Cervical Self-Sampling Program in Kenya.

**Methods:**

A multimethod approach, involving participatory action research, scenario based planning, and phenomenology, was applied in conjunction with two tools to identify relevant factors (negative or positive) that could impact Cervical Self-Sampling Program implementation. A total of 107 stakeholders participated in interviews, focus groups, workshops, and informal interactions. Content analysis, an affinity exercise, and impact analysis were used to analyze data and develop robust strategic directions and supporting implementation strategies.

**Results:**

A total of 57 factors thought to impact the implementation of the Cervical Self-Sampling Program were identified and grouped into 13 thematic categories. These themes were instrumental in developing 10 strategic directions and 22 implementation strategies deemed necessary to implement a technically viable, politically supported, affordable, logistically feasible, socially acceptable, and transformative Program.

**Conclusions:**

This study made three conclusions: 1) there is political will and a desire to improve cervical screening across Kenya, but in a period of dynamic change resources are constrained; 2) implementing the Program in urban/rural settings is logistically feasible, but the majority of Kenyan women could not afford screening without some form of a subsidy, and 3) self-sampling is perceived to be much more socially acceptable than the current Pap screening process. The Chaguo Letu study went beyond the traditional strategy development process of determining “what” needs to do done by describing in detail “how” the Program should be implemented to be relevant and accessible to all Kenyan women at risk of cervical cancer. This work could potentially facilitate communities of practice and knowledge sharing when addressing other types of health decisions in other low resource settings beyond Kenya.

## Background

Over three decades ago, Susman & Evered [1] spoke about a crisis in organizational science in which scholarly research did not relate to the real world of practicing managers. This situation has persisted over time with numerous health policy makers and researchers expressing their concerns about the limitations of research contributing to evidence based decision/policy making [2–5]. Some organizations have emphasized the importance of building the capacity of policy and decision makers to effectively access and use relevant research findings [6–8]. However, progress has been slow; many health organizations have little capacity to operationalize research to strengthen health systems [2, 9, 10].

Some scholars believe that health leaders would achieve greater benefit from science if the research agenda addressed contextual and implementation issues and if researchers and other users collaborated to define the research agenda, allocate resources, and implement the findings [11, 12]. The Participatory Action Research (PAR) methodology advocates for the integration of research into practice and broad-based participation in the implementation of a locally driven agenda [13, 14]. By including local experts (with the required tacit, explicit, and cultural knowledge) in the decision-making process, ownership of decisions could be strengthened, thereby increasing the probability that they will be acted upon [6, 15–17].

This study’s aim was to determine how local decision makers could apply a multimethod approach to make good strategic decisions on how to implement a Cervical Self-Sampling Program (CSSP) in Kenya. This is of particular importance given that 12.92 million women in Kenya aged 15 years and older are at risk of cervical cancer (CC). Since less than 3.2% of women aged 18–69 years are screened, it is not surprising that 4802 Kenyan women are diagnosed with CC annually and 2451 die from the disease [18, 19]. Minimizing the risk of making bad CSSP implementation decisions was mitigated by implementing the Scenario Based Planning (SBP) method [20] (to ensure a logistically feasible program) and the Existential Phenomenology (EP) method [21] (to promote a culturally sensitive program that would be adopted by Kenyan women).

Several studies in the United States [22–24]; Netherlands [25]; Canada [26]; India, Nicaragua and Uganda [27], and Kenya [28], have investigated specimen self-collection to determine if it can increase the effectiveness and coverage of CC screening. These studies generally agree that self-sampling could be a useful component of screening programs, especially where women have limited access to screening, or are reluctant to undergo screening due to cultural beliefs. Jones et al. [22] reported that 96% of low-income women were very/somewhat comfortable collecting their own sample. Despite organized outreach in the Netherlands, over 30% of women do not respond to screening invitations involving clinician specimen collection [25]. In a rural community in Newfoundland Canada, cervical cancer screening increased by 15.2% where self-collection was available [26]. Rositch et al. [28] showed that 82% of Kenyan women would feel comfortable using a self-sampling device. Although all of these studies suggest that self-sampling could be useful to increasing overall coverage of cervical cancer screening, there is limited evidence in the literature guiding decision makers on how to implement an appropriate CSSP in low-resource settings. This research study investigated how to fill this gap.

## Methods

**Fig. 1:**
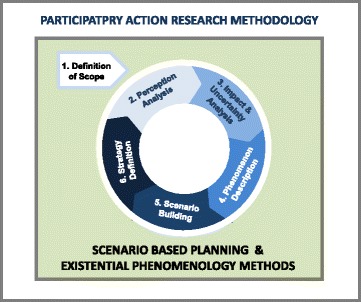
Chaguo Letu project multimethod study design

The study design (Fig. 1) for this multimethod qualitative research study included three main components: the Participatory Action Research (PAR) methodology [13, 14], Scenario Based Planning (SBP) method [20], and Existential Phenomenology (EP) method [21, 29, 30].

Each of these three components made a unique contribution to enabling local decision makers to collectively make informed, practical, culturally sensitive strategic decisions on how best to implement a CSSP in Kenya.

### Participatory Action Research Methodology

The PAR methodology philosophically grounded the Project Team [the Principle Investigator (PI), Project Management Team (PMT), and Local Decision Influencing Participants (LDIP) members] as they progressed through four study phases: 1) teaming, 2) diagnosing and planning, 3) acting and reflecting, and 4) specifying learning [13, 14]. The Project Team refined three PAR principles to influence their behaviour and guide and direct them throughout the study: 1) collaborative participatory action to solve problems, 2) linking scientific understanding to social action, and 3) co-construction of knowledge through research to influence social improvement [13–15, 31–33]. The degree to which these principles were demonstrated was evaluated by the Project Team at the conclusion of each of four workshops with the use of a confidential evaluation tool. To facilitate compliance with the PAR principles, the PI embraced the concept of power sharing by providing the education and mentoring needed to enable study participants to act as co-researchers, build their decision-making capacity, and take responsibility for co-owning the research process.

### Scenario Based Planning Method

The SBP method, with its perception analysis, scenario visualization and impact analysis tools and techniques, was applied to minimize the risk of making bad decisions regarding the design of the CSSP. With this objective in mind, Wulf, Meissner & Stubner’s [20] SBP method (Fig. 1) was adapted for this study. The method included six steps:

1. Definition of scope included validation of the project goals and objectives, scope of data collection and analysis, time frame, and team membership.
2. Perception analysis involved analyzing Project Team members’ and external subject matter experts’ perceptions, assumptions, and beliefs.
3. Trend and uncertainty analysis identified the most important driving forces (factors) that could affect the implementation of a CSSP. Factors were ranked by their level of importance/potential impact and their degree of uncertainty to identify those that were most crucial to development of CSSP implementation strategies.
4. Phenomenology description involved determining the essence of women’s perceptions of CC and self-sampling, i.e., identifying those factors that would predispose a woman to adopt or reject collecting her own cervical sample.
5. Scenario building involved creating potential scenarios that described different future states for the CSSP in Kenya. Additional data for each potential scenario was collected to produce plausible descriptions of how a CSSP could be implemented, followed by an impact analysis to determine the consequences of each scenario.
6. Strategy definition included development of the CSSP strategic directions and supporting implementation strategies.

### Existential Phenomenology Method

The EP method was applied to investigate the existential meaning of cervical self-sampling and determine the social acceptability of self-sampling by Kenyan women. Understanding what would influence adoption and rejection of the CSSP was crucial to its design. The EP approach [21, 29, 30] consisted of six steps:

1. The phenomenological research objective was developed and examined as to its fit with the study design.
2. The Principle Investigator (PI), Project Management Team (PMT) and Local Decision Influencing Participant (LDIP) members decided to be consistent with the PAR methodology and operate as co-decision makers. The PI did not bracket her own lived experience with CC, as this would diminish the tight relationship being encouraged with co-participants [21, 30, 34]. PI engagement in discussions to gain contextual understanding and meaning about cervical self-sampling took precedence over attempts to bracket preconceptions [35].
3. The PI developed a discussion guide in collaboration with the PMT to ensure that sensitivity to local values and culture were addressed when conducting semi-structured interviews and focus groups with urban and rural women [21, 30]. The PI (through a local PMT member acting as a translator) asked each participant to provide a description of their conscious experience pertaining to CC screening, and their thoughts on cervical self-sampling. Participants were asked to provide their perspectives in response to two statements: tell me about your experiences with CC, and tell me about those things (good or bad) that you think would have an impact on the implementation of a cervical self-sampling program. During the interviews and focus groups, the PI (through the translator) articulated her interpretation of what participants described to better validate the participants’ meaning. This process of concurrent member validation aided in ensuring that the PI appropriately understood the participants’ comments [36, 37].
4. The PI conducted a critical review of the transcripts of each participant interaction and highlighted any significant statements and quotes, referred to as units of significance. These 14 units of significance were clustered into five themes and into two broad categories. This process showed the confluence of all participants’ views under different perspectives, i.e., the decision variables and factors influencing urban and rural women’s decisions to adopt or reject cervical self-sampling [21].
5. The units of significance and themes were used by the PI to write two textural descriptions of women’s perceptions of cervical self-sampling; one for an urban setting and one rural. The composite descriptions articulated the essence of how women decided whether to adopt or reject cervical self-sampling [21].
6. To ensure data quality regarding the composite descriptions, the PI reviewed the outputs from the phenomenological assessment with PMT and LDIP members in a workshop setting and requested their feedback. Given that some of the female members had also been interviewed, this process provided an additional opportunity for member validation, also referred to as member checking. Upon review of these outputs and engaging in open dialogue, the Project Team collectively decided on how this information pertaining to social acceptability would be addressed in the design of the CSSP. This power sharing relationship, in which decisions were made collectively, contributed to a more comprehensive CSSP design [36–39].

Applying any one of these methods on its own would not have enabled the Project Team to design a relevant, sustainable CSSP. The integration of these three components contributed to the co-creation of the knowledge required to design the best CSSP solution. PAR promoted local collaborative decision making behaviour, SBP enabled the design of a logistically feasible CSSP taking all factors into consideration, and EP ensured that the CSSP would be socially acceptable.

### Multimethod Data Collection and Analysis

To ensure efficacy, credibility and transferability of study results, a multimethod approach to data collection and analysis was applied [29, 40–42]. Investigating multiple constructs (i.e., cervical self-sampling, decision-making, and strategy development) requiring different analysis efforts supported the rationale for applying a multimethod approach [42]. For these reasons, data collection and analysis methods, which are aligned with the PAR methodology [13, 14, 32], SBP method [27, 43], and EP method [21, 29, 30], were incorporated into this study.

**Fig. 2:**
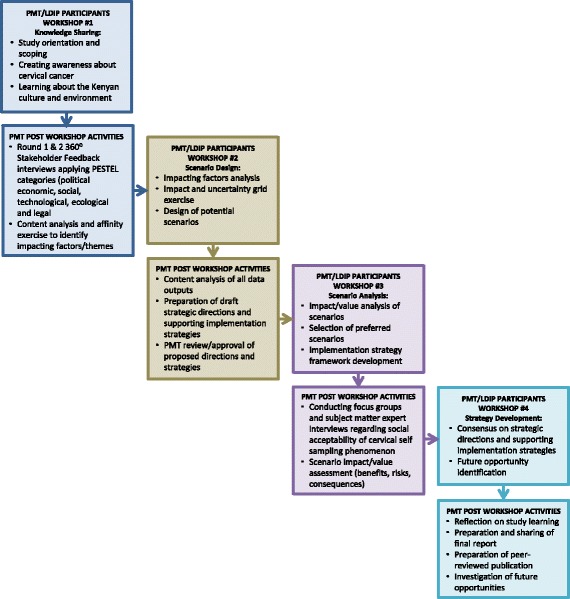
Iterative multimethod data collection and analysis approach

Consistent with the qualitative research paradigm of constructivism [44] and PAR [14, 32], data collection and analysis was a collaborative, participative, reflective and iterative process. Repeated rounds of data collection and analysis took place before, during and after four Workshop events. Figure 2 shows which activities were carried out during each workshop by the PMT and LDIP members, plus the subsequent data collection and analysis PMT members conducted after each workshop. These outputs were then brought forward to the next workshop for everyone’s input and validation.

Data was collected through: formal interviews, focus groups, workshops, and informal interactions. Forms of data collected included: audio tape recordings of interviews, focus groups and workshops, transcriptions of the recordings, and textual descriptions of observations in a personal journal.

Applying the principle of triangulation [45], a series of analytic processes were conducted in support of four Workshops (Fig. 2). Initially, data analysis focused on conducting content analysis [46] of outputs from SBP 360^0^ Stakeholder Feedback interview transcripts to identify factors (negative or positive) that could impact implementation of a CSSP. The similarity between factors was then assessed as part of an affinity exercise [47] to categorize factors into thematic groupings. Following the design of potential self-sampling scenarios, the phenomenology method of analysis [21, 29, 30] was applied to identify situational and emotional units of significance for adoption of cervical self-sampling phenomenon. As a last step, a final round of content analysis, aggregating all study findings, was conducted to identify CSSP strategic directions and supporting implementation strategies.

### Study Participants

Brock University, Action Africa Help International (AAHI), and the University of Nairobi linked their resources and scientific backgrounds, with LDIP’s, to ensure the design of a culturally sensitive, practical approach for cervical self-sampling. With this goal in mind, AAHI applied a purposeful, criterion sampling approach to select the LDIP’s based on: a) existing relationships, b) recommendations from respected key informants, c) fit with desired characteristics, e.g., familiar with cervical cancer, possess technical and/or content expertise, and d) ability to influence solution adoption.

Nine PMT members along with 13 LDIP’s comprised the Project Team (PT), 18 of which were Kenyans (Table 1). Although this was a large team, it proved to be manageable and was necessary to provide confidence in the study results; it ensured that sufficient numbers of relevant stakeholders were engaged in CSSP design during workshops, and promoted future adoption of the CSSP. Other urban participants included eight subject matter experts. In addition, 97 rural participants from four Kenyan counties provided valuable input in the consultation process regarding the social acceptability of the CSSP (Table 1). Determining which counties to focus on and which interview and focus group participants to include were based on their proximity to Nairobi, plus AAHI and University of Nairobi’s relationships that could be leveraged.

**Table 1:** Study participants

Location	Demographics
Urban Participants—Total of 30 (21 women and 9 men)	
City of Nairobi in Nairobi County	9 Project Management Team members: Action Africa Help International employees (2 women and 2 men); non-Kenyan researchers (1 woman and 2 men); a Kenyan consultant/translator (1 woman), and a Grand Challenges Canada representative (1 woman)
City of Nairobi in Nairobi County	13 Local Decision Influencing Participants: Ministry of Health leaders (3 women, 1 man); public health laboratory leaders (2 men); private laboratory leader (1 man); county health services leader (1 women); university professor (1 woman); NGO leader (1 man); practicing gynaecologists (3 women)
City of Nairobi in Nairobi County	8 subject matter experts: all professional middle-aged women such as: clinicians (3), pharmacists (2), administrators (3)
Rural Participants—Total of 97 (94 women and 3 men)	
Ndumago Community Unit, Kiambu County	11 female Community Health Volunteers (CHV’s) and 3 male CHV’s
Ole Sere Village, Narok County	20 village women
Sekenani Village, Narok County	12 village women
Tala Village, Machakos County	47 village women
City of Thika, Kiambu County	3 female CHV’s and 1 female health system administrator

The Project Team chose to name this study the Chaguo Letu Project. Chaguo Letu is a Swahili term that means “our choice”.

## Results

### Scenario Based Planning Results

**Fig. 3:**
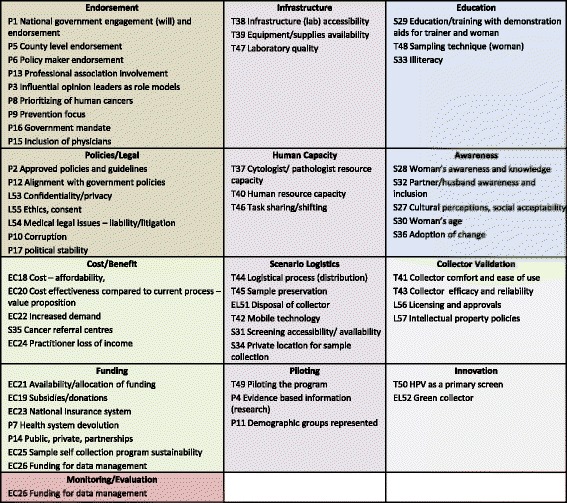
CSSP Thematic Factor Categories

Applying content analysis [46] on the Round One 360° Stakeholder Feedback results identified 57 factors thought to have an impact (positive or negative) on CSSP implementation. Grouping these factors as part of the affinity exercise [47] produced 13 thematic categories (Fig. 3).

**Fig. 4:**
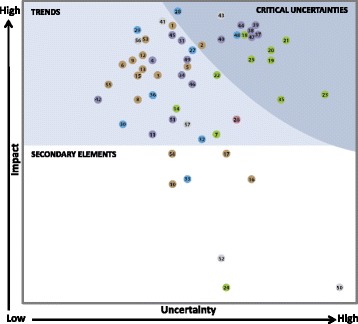
CSSP Impact and Uncertainty Grid Results—Legend

Figure 4 shows the results of plotting the 57 factors on the Impact and Uncertainty Grid [48]. Factors with high impact ratings and high to moderate uncertainty ratings, were identified as the critical uncertainties serving as the basis for CSSP strategic directions. Factors that had high to moderate impact ratings and moderate to low uncertainty ratings were incorporated within the CSSP supporting implementation strategies. The remaining factors were identified as secondary elements of least importance.

### Social Acceptability Analysis Results

A total of 107 participants (97 rural and 10 urban) responded to six binary questions asked during five focus groups and 10 interviews (Table 2). The purpose of these questions was to provide background information on the current level of CC knowledge and experience, plus social acceptability of self-sampling by urban and rural Kenyan women. The tabulation of responses for question three shows that all but 10 rural women found cervical self-sampling socially acceptable.

**Table 2:** Focus group/interview guide question results

Focus group & interview questions	Response tabulated	Total rural participants 97	Total urban participants 10
1. Do you know what cervical cancer is?	Yes	32	10
2. Have you ever been screened?	Yes	13	9
3. Would you be willing to collect your own sample in a private place?	Yes	87	10
4. Would you prefer to collect your own sample in your home?	Home	24	6
5. Would you prefer to collect your own sample in a clinic?	Clinic	19	1
6. Would you prefer to collect your own sample in your home or in a clinic?	Both	54	6

### Phenomenology analysis results

**Fig. 5:**
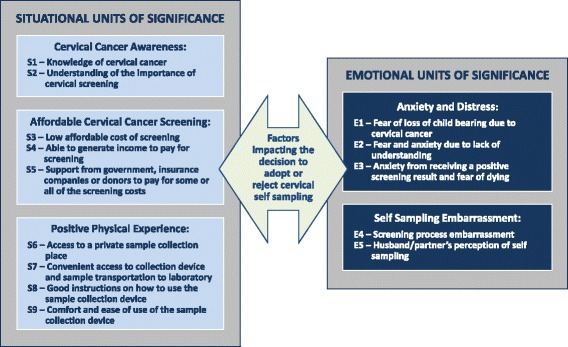
Situational and emotional units of significance influencing cervical self-sampling adoption or rejection

The phenomenology analysis [21] conducted by four PMT members during two working sessions reduced all interview/focus group statements and quotes into 14 units of significance, followed by clustering these into five themes. Following in-depth reflection on the units of significance by four PMT members, it became apparent that the clusters had an affinity to two main broad categories. Three thematic clusters represented units of significance dealing with situational variables, while two themes emphasized emotional variables (Fig. 5). Examples of quotes (by theme) include the following:

- Cervical cancer awareness: “If I’m not informed and don’t know—it doesn’t help you. In this case if you don’t know it will hurt you.”
- Affordable cervical cancer screening: “If too much cost then need to choose between paying for food or the test. The test should cost no more than 20 shillings. Many poor women can afford nothing.”
- Positive physical experience: “Some women might not get an adequate sample, or get it (collector) in the wrong place if not well understood on the iPap usage—it is simple but needs some directions in the local language.”
- Anxiety and distress: “…you send a text message and say your results are back and we would like you to visit a health facility—that can crush a woman. The fear of cervical cancer is a bit high up.”
- Self-sampling embarrassment: “…in rural areas women don’t even want to touch their private parts, so for someone else doing it, it’s far worse than them doing it themselves.”

Figure 5 shows that both situational and emotional variables impact the decision to adopt or reject cervical self-sampling. Situational units of significance could be described as precursors to initiating the decision to adopt or reject cervical self-sampling. If these variables are not mitigated, a woman would automatically reject the option of self-sampling. A woman would need to know about cervical self-sampling, can afford the test, and have the physically means and knowledge as to how to collect her own cervical sample, to proceed to the step of deciding if this is something she wants to do. Providing these situational units of significance are addressed, a woman is then in the position to contemplate how she “feels” about cervical self-sampling. This is when emotional units of significance come into play. The level of anxiety and distress she is feeling, how embarrassed she would be to collect her sample, and her husband’s or partner’s perception about this test could influence her decision to either adopt or reject cervical self-sampling.

### Scenario Impact Analysis Results

**Fig. 6:**
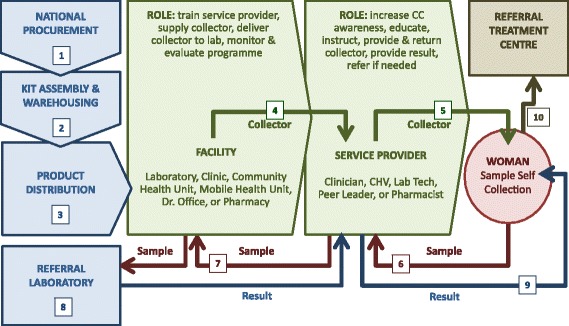
CSSP generic implementation scenario

Data outputs from previous analyses provided the input for the impact analysis of each proposed scenario [49, 50]. This included identifying the risks and consequences of implementing each scenario, and the commonalities and differences between scenarios. This exercise led to the Project Team developing a generic CSSP scenario involving 10 steps (Fig. 6).

**Fig. 7:**
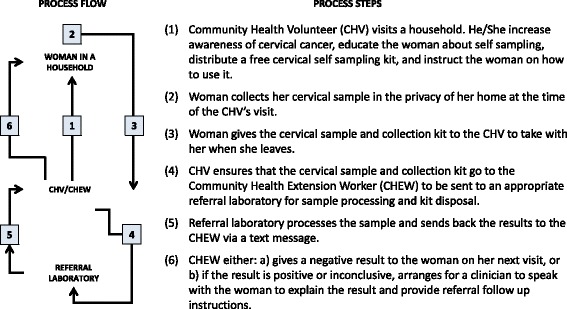
CSSP community health volunteer scenario

**Fig. 8:**
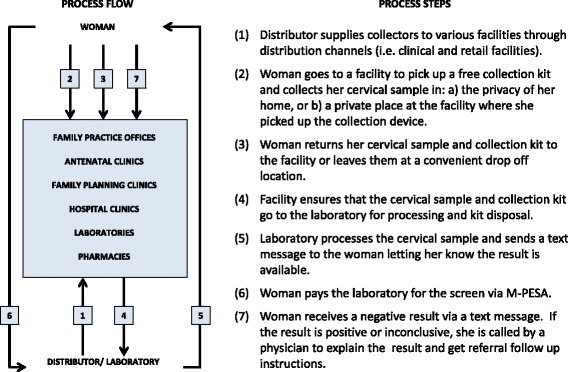
CSSP urban clinic/retail scenario

In addition, two unique applications were created—one to show how the CSSP could be implemented within a retail/clinical urban environment (Fig. 7), and one to demonstrate its application as a Community Health Volunteer scenario in rural settings (Fig. 8).

Content analysis of all aggregated data analysis outputs provided a mechanism to: translate data into meaningful information, gain insight through assessing scenario impact, construct new knowledge, and subsequently inform the Project Team to make appropriate CSSP implementation choices [51]. As a result, the Project Team was well informed and able to finalize the CSSP strategic directions and supporting implementation strategies.

### CSSP Strategic Directions and Supporting Implementation Strategies

After extensive data collection and analysis of all factors identified, the Project Team determined that there were 10 recommended strategic directions, along with a total of 22 supporting implementation strategies that collectively provide sufficient clarity on how to successfully implement the CSSP.

Strategic Direction #1—Collector Validation: to acquire proof that the cervical self-sampling device has been successfully validated, has licencing and regulatory approval, and is safe and appropriate to procure and use within Kenya.

1.1 To clarify and validate with product developers, Kenya Ministry of Health, and relevant regulatory bodies, the following:
  - Adequate level of collector comfort, and ease of use, when a woman is using the device to collect her own cervical sample.
  - Any potential side effects that a woman could experience as a result of collecting her own sample.
  - Collector efficacy and reliability to collect an adequate sample (5000 cells or more), when used by a woman, as compared to existing collection devices used by a physician to collect a cervical sample.
  - Collector kit preservative vial is of sufficient quality to preserve the liquid sample in local climatic conditions.
  - Existing collector licensing agreements and product approvals meet National regulatory requirements regarding procurement and use in Kenya.
1.2 To investigate funding opportunities to conduct a validation of the self-sampling device by the University of Nairobi to determine the device’s ability to screen for multiple diseases using the same cervical sample.

Strategic Direction #2—Endorsement**:** to gain the political will needed, and to ensure comprehensive endorsement of the CSSP (at all levels) by relevant stakeholders and key opinion leaders, from the outset of the Program.

2.1 To design and implement a stakeholder engagement process, governance structure, and decision model that will incorporate all relevant stakeholders and key opinion leaders to:
  - Influence adoption and endorsement of the CSSP.
  - Ensure the ongoing focus on CC as a priority amongst human cancers.
  - Support a continued focus on the prevention of CC.
  - Address any implementation issues and sensitivities displayed by stakeholder groups.

Strategic Direction #3—Policies/Legal: to develop and approve all relevant policies and procedures for implementation of the CSSP, along with any related legal issues, and to ensure alignment of these CSSP policies with current government policies, standards, guidelines and regulations.

3.1 To undertake a comprehensive policy and procedure development, approval, and continuous updating process that will address all aspects of effective and appropriate implementation and management of the CSSP and ensure alignment of CSSP policies with existing government policies. This should include the following areas:
  - Logistical implementation policies, standard operating procedures, guidelines, standards, and regulations for all aspects of the CSSP logistical process.
  - Confidentiality/privacy policies and procedures to protect the rights of women participating in the CSSP and to ensure that their data is managed appropriately when being used for secondary purposes.
  - Ethics protocols and consent policies that go beyond implied consent in both the public and private sectors.
  - Potential medical liability/litigation policies dealing with the determination of if/who would be liable if the sample result is a false negative.
  - Intellectual property policies regarding ownership of data outputs resulting from the application of the collection device; these policies must be comprehensive, clear, and supported by the Kenya Ministry of Health.

Strategic Direction #4—Cost/Funding: to ensure adequate funding to pilot and implement the CSSP, and to develop/implement funding strategies that will ensure that the cost of the CSSP is low and affordable by Kenyan women at all economic levels. This will enable all women to access the Program and realize the benefits of detecting cervical disease early.

4.1 To ensure that adequate funding has been assigned at the outset of the CSSP to enable both piloting and implementation of all components of the Program. Potential sources for CSSP investment could encompass both National and County governments, along with other private and public entities.
4.2 To explore, develop and implement a variety of costing/funding options to promote CSSP affordability, while delivering sufficient benefit.

Strategic Direction #5—Infrastructure/Human Capacity: to develop and make available the infrastructure that is required, along with sufficient numbers of appropriate, qualified resources to support the CSSP throughout both urban and rural/community environments in Kenya.

5.1 To develop and implement a cost effective implementation plan addressing the current gaps that would impact the timely, successful implementation of the CSSP. The objectives of the implementation plan are to:
  - Increase access to quality laboratory facilities with available, appropriate equipment and sufficient supplies to address the anticipated increased demand for sample processing, resulting from the implementation of the CSSP.
  - Address the training requirements and capacity needs for all human resources that will be involved with cervical self-sampling.
5.2 To develop role descriptions and responsibilities for all of the individuals that will be involved with the CSSP.

Strategic Direction #6—Scenario Logistics—Piloting and Implementation: to initially pilot and validate the logistical feasibility of the CSSP scenario design, followed by refinement of the design, and finally its implementation across Kenya.

6.1 To engage in public/private partnerships to pilot and implement an accessible and affordable Program across Kenya. The objectives of this strategy include:
  - To create a consortium of public and private entities that collectively will be able to raise the necessary investment and resources needed to effectively pilot and implement the CSSP in a timely fashion.
  - To acquire the best possible quality and pricing for the collection kit components.
  - To broaden distribution channels into hard-to-reach areas in Kenya.
  - To create local jobs.
6.2 To conduct a research study to pilot and validate the initial CSSP design in three settings: 1) an urban clinical setting, 2) a retail pharmacy environment, and 3) a rural community health volunteer setting. The logistical strategies to be piloted and validated include the following:
  - Procurement: To determine the acceptability and viability of a National centralized procurement process in which separate elements of the collection kit (i.e., sample collection device, preservation vial, and customized instructions for use document) are purchased in bulk by a consortium (sanctioned by government) and then assembled and warehoused centrally in Nairobi, Kenya by a wholesaler.
  - Distribution: To apply a segregated approach to distribution in which various facilities (e.g., clinical settings, retail environments, pharmacies and laboratories) can purchase the collection kit at a standard cost from the wholesaler, and then offer it to their clients applying different pricing strategies according to a woman’s ability to pay (see section “C. Affordable”).
  - Cervical sample collection: To trial: 1) educational materials prepared for service providers, 2) educational materials prepared to increase a woman’s awareness of CC, 3) the applicability of the instructions for use document, and 4) package handling instructions. To identify which locations are most suitable for collection of the cervical sample, i.e., private, clean, and comfortable.
  - Cervical sample and collection kit transportation: To trial different “unique identifier” approaches to ensure that a woman’s sample is appropriately linked to her demographic information. To apply mobile technology to collect this demographic data and link it to the woman’s result. To identify various mechanisms to “drop off” the cervical sample in an appropriate, convenient and confidential location. To trial various sample pick up schedules that will ensure timely transportation to a referral laboratory as per the guidelines associated with the climatic requirements for the preservation of the sample in the vial.
  - Disposal of cervical sample collection kit: To refine the disposal process of the collection kit to ensure that: 1) the collection device is appropriately packaged following its use, and 2) that the collection device is returned to the laboratory (with the cervical sample) to be properly disposed of (according to current policies for disposal of potentially bio hazardous material). The recommendation is to delay processing of the sample until the woman returns the collector.
  - Sample processing payment (only applicable when a woman will be paying for the processing of her cervical sample): To trial the applicability of: 1) the laboratory notifying the woman through a text message that the results of her screen are available and requesting the woman to pay for the test, and 2) the viability of the payment transaction being made via M-PESA (a mobile phone application).
  - Results delivery: To trial the process by which a woman would receive the results of her cervical screen. The recommendation is that a negative result could be sent via a text message, however, if the result is positive or inconclusive, the woman could be called by a clinician to explain the result and it’s implications. During this person-to-person interaction, the clinician could provide referral follow up instructions as needed. The acceptability of being notified of a positive result via text message will also be trialed.
6.3 To provide some form of benefit to the communities in return for their participation in the pilot studies, such as providing them with early access to new screening technologies once they are available.
6.4 To collect data (i.e., demographic, financial, administrative and clinical) during the course of the CSSP pilot to enable refinement of the Program and ensure it is logistically feasible, affordable and socially acceptable. This evidence is also intended to demonstrate the economic value (i.e., cost/benefit analysis) of fully implementing the CSSP.

Strategic Direction #7—Culture/Awareness: to design and implement culturally sensitive approaches to increase awareness about CC and the CSSP.

7.1 To develop and implement a timely (i.e., just prior to the CSSP pilot study), comprehensive, and culturally sensitive CSSP awareness campaign that will address/incorporate the following:
  - Cultural norms associated with a woman’s age, husbands making health decisions for their wives, illiteracy, geographic location, level of education, poverty level, social status, etc.
  - Leveraging existing CC awareness campaigns to emphasize the importance of cervical self-sampling for improved access and early disease detection.
  - Application of mobile technology and social media where and if possible to push user-friendly information about CC and self-sampling across Kenya.
  - Application of creative distribution channels to disseminate information about CC and self-sampling.

Strategic Direction #8—Education: to design and implement (in collaboration with government and relevant educational bodies and professional associations, such as the Kenya Medical Women’s Association) effective cervical self-sampling educational and instructional materials that will provide both trainers and women with the knowledge and skills they need to participate in the CSSP.

8.1 To purchase and/or develop training aides such as the following:
  - Reproductive health system visual aids to demonstrate where the cervix is in relation to other organs (simple graphics, clear plastic models, videos that can be used with various mobile technologies).
8.2 To develop a Trainer educational kit that would include the following:
  - Background information and teaching aids on what CC is and how you get infected with it.
  - Scripts on what to say to a woman and her husband/partner about CC and cervical self-sampling.
  - How to demonstrate and provide instruction (with a self-sampling device and teaching aids) to a woman (and her husband/partner) to collect her own cervical sample.
8.3 To develop a Cervical Self-Sampling Instruction Kit for the trainer to use (via different media) in the presence of the woman (and her husband/partner) that would include the following:
  - Pictorial information about CC.
  - Information on why and how to collect a cervical sample.
  - Step-by-step graphical and narrative (in the appropriate language) instructions for use to: 1) prepare for sample self-collection, 2) collect the sample, 3) reassemble all kit components into provided packaging with required demographic information, and 4) drop off the sample for pickup and transportation to the laboratory.

Strategic Direction #9—Innovative, Sustainable Cervical Cancer Screening/Value Proposition: to apply out-of-the-box thinking and investigate innovations, which have the potential to transform how women in Kenya get screened and tested for CC.

9.1 To investigate the viability of implementing innovations that would assure the sustainability of a CC screening program in the future. At a minimum, these could include the following:
  - To solicit funding for, and validate, the capability to screen for multiple diseases from one cervical sample collected with a self-sampling device.
  - To investigate the Kenyan implications of: 1) conducting HPV screening as a primary screen for CC, and 2) HPV vaccination.
  - To determine the viability and timing of implementing a biomarker based point of care screening device that would enable CC screening at the point of care.
9.2 To initially investigate the viability of conducting an analytic value proposition for implementing both the CSSP and a biomarker based point of care screening option to radically transform how women in Kenya could be screened for CC in the future. Providing this is doable, the scope of the value proposition could include the following:
  - Costs and outcomes related to the operation of current CC screening and treatment programs.
  - Costs and potential benefits related to implementing the CSSP.
  - Costs and potential benefits related to implementing point of care biomarker based CC screening in combination with the CSSP.

Strategic Direction #10—Impact Management: to develop and implement a CSSP impact management process that would incorporate data management components to facilitate Program monitoring, evaluation, and reporting, plus leveraging of CSSP data outputs for secondary use.

10.1 To ensure adequate funding and human resources are made available to support CSSP data management, i.e., data generation/collection, data manipulation and analysis, information dissemination, and data/information storage.
10.2 To design and implement a CSSP impact management process that would address the following:
  - Ongoing impact assessment, i.e., monitoring and evaluation of CSSP inputs and outcomes.
  - Routine reporting of CSSP impacts (positive and negative).
10.3 To investigate how CSSP data outputs could be collated with other existing health information databases and repositories to support effective health management.
10.4 To collaborate with government, clinical and academic partners to facilitate the ethical, confidential use of CSSP data for secondary purposes.

## Discussion

This study supported the findings of other studies showing that women are willing to collect their own cervical sample. All but 10 out of 107 Kenyan women participating in this study found cervical self-sampling socially acceptable. This is in line with studies conducted by Rositch et al. [28] in Kenya in which 82% of women were willing to self sample, and the works of Jones et al. [22] in which 96% found self-sampling comfortable and 79% would prefer to self sample the next time they were screened. Although this finding was important, the key contribution of this study was the determination of “how” to effectively implement a CSSP in Kenya.

The collaborative and participatory efforts [15] of the Chaguo Letu Project Team were instrumental in the development of 10 strategic directions and 22 supporting implementation strategies that need to be addressed to implement a cervical self-sampling program; one that has the greatest potential to be technically viable, politically supported, affordable, logistically feasible, socially acceptable, and transformative. This study supports the premise that all known variables need to be taken into consideration when planning for the implementation of a CSSP. Dealing with just some, rather than all, significantly jeopardizes success [52].

The Chaguo Letu study went beyond the traditional strategy development process of determining “what” needs to do done by describing in detail “how” the CSSP needs to be implemented, and by identifying exemplary practices and enablers of predictive and prescriptive decision-making [51, 53, 54]. The application of the 360° Stakeholder Feedback tools provided a structured, comprehensive approach for participants to predict which factors would have an impact, and the underlying uncertainties regarding CSSP implementation. Impact analysis of multiple implementation scenarios enabled decision makers to make informed decisions and prescribe the preferred option [53, 55], allowing participants to: 1) translate the data into meaningful information, 2) gain insight through assessing impact, 3) construct knowledge, and 4) subsequently inform project participants to make appropriate choices [51, 56].

Applying a multimethod approach [45] using the PAR methodology with the SBP method and analytic tools and techniques helped the Project Team make strategic, prescriptive decisions [57]. Having a combination of one-on-one interviews, focus groups, and group participation at workshops, proved to be a successful approach to support collaborative decision-making. Incorporating the EP method delineated the phenomenon of cervical self-sampling and informed the SBP process with the cultural knowledge needed to design a socially acceptable self-sampling scenario [58].

Since review of the literature did not identify any similar studies to substantiate the application of this multimethod approach in other settings, the Project Team is recommending that further research be conducted replicating this approach in other low to middle income countries.

## Conclusions

This study made three conclusions pertaining to the implementation of a CSSP in Kenya: 1) there is political will and a desire to improve many aspects of CC screening across Kenya, but in a period of dynamic change resources are constrained [59]; 2) implementing a CSSP in urban/rural settings across Kenya is logistically feasible, but the majority of Kenyan women could not afford CC screening without some form of a subsidy [60], and 3) the self-sampling approach is perceived to be much more socially acceptable than the current Pap screening process [2]. Unfortunately, the consequence of implementing the CSSP would increase the demand for screening and therefore have a significant impact on the need for infrastructure, human resources, and treatment investments. The current Kenyan health system could not manage this additional burden independently at this time. This study shows a significant need for Public/Private Partnerships (PPP) to share the investment costs associated with implementing a CSSP, which is in line with the PPP Act [61].

Given the large investments required to implement the CSSP, the Project Team is recommending investigation of coupling self-sampling with biomarker-based point-of-care testing (currently under development) to radically transform the CC screening process from beginning to end [62]. This approach could minimize the need for increased human resources and infrastructure strengthening [63].

## Abbreviations

CC: Cervical cancer
CHV: Community health volunteer
CSSP: Cervical self-sampling program
EP: Existential Phenomenology
HPV: Human papillomavirus
LDIP: Local decision influencing participant
PAR: Participatory action research
PI: Principle investigator
PMT: Project Management Team
PPP: Public, private partnership
SBP: Scenario based planning
